# Tracking Charge Carrier Paths in Freestanding GaN/AlN Nanowires on Si(111)

**DOI:** 10.1021/acsami.4c10179

**Published:** 2024-09-19

**Authors:** Juliane Koch, Patrick Häuser, Peter Kleinschmidt, Werner Prost, Nils Weimann, Thomas Hannappel

**Affiliations:** † Department of Mathematics and Natural Science, Institute for Physics, Fundamentals of Energy Materials, Ilmenau University of Technology, Ilmenau 98693, Germany; ‡ Components for High Frequency Electronics (BHE), University of Duisburg-Essen, Duisburg 47057, Germany

**Keywords:** GaN, nanowire, multitip scanning tunnelling microscopy, MOVPE, III−V semiconductor

## Abstract

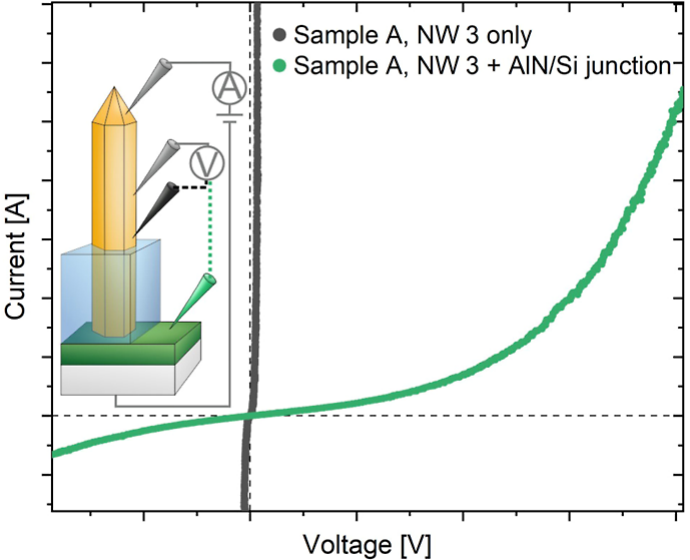

Functional and abundant substrate materials are relevant for applying all sophisticated semiconductor-based device components such as nanowire arrays. In the case of GaN nanowires grown by metalorganic vapor phase epitaxy, Si(111) substrates are widely used, together with an AlN interlayer to suppress the well-known Ga-based melt-back-etching. However, the AlN interlayer can degrade the interfacial conductivity of the Si(111) substrate. To reveal the possible impact of this interlayer on the overall electrical performance, an advanced analysis of the electrical behavior with suitable spatial resolution is essential. For the electrical investigation of the nanowire-to-substrate junction, we used a four-point probe measurement setup with sufficiently high spatial resolution. The charge separation behavior of the junction is also demonstrated by an electron beam-induced current mode, while the n-GaN nanowire (NW) core exhibits good electrical conductivity. The charge carrier-selective transport at the NW-to-substrate junction can be attributed to different, local material compositions by two main effects: the reduction of Ga adatoms by shadowing of the lower part of the NW structure by the top part during growth, i.e. the protection of the pedestal footprint from Ga adsorption. Our combination of investigation methods provides direct insight into the nanowire-to-substrate junction and leads to a model of the conductivity channels at the nanowire base. This knowledge is crucial for all future GaN bottom-up grown nanowire structure devices on conductive Si(111) substrates.

## Introduction

III–V semiconductor nanowires (NW) form fundamental building blocks for a wide range of applications in electronic and optoelectronic devices. By specifically adjusting the material composition, the electronic structure can be tuned according to the desired application.^1^ In particular, the GaN-based material system has attracted much attention in recent years.^2,3^ GaN NWs can be used in a variety of applications such as in LASERs,^4−6^ field effect transistors,^7−9^ solar cells,^10−12^ sensors,^13,14^ photodetectors,^15−18^ LEDs,^19−21^ or photoelectrochemical water splitting cells.^22,23^ GaN is preferably grown in *c⃗* direction, exhibiting strong electrical polarization fields that cause a long carrier lifetime for GaN LEDs^24^ and large offset voltages for resonant tunneling diodes.^25,26^ Conversely, the multiquantum well structures grown on the *m*-plane side facets of a *c⃗* grown GaN NW are free of polarization fields enabling LEDs with high modulation speed.^20^

GaN-based electronics and optoelectronics frequently use Si(111) substrates. Despite the high lattice mismatch, it is used as a low-cost, large-area, and thermally and electrically conductive substrate.^27,28^ However, due to the high GaN growth temperature, Si may diffuse from the substrate into the GaN, and Ga may diffuse from the GaN into the Si substrate. The interaction between GaN and Si already occurs at low growth temperatures.^29^ The latter process is known as Ga-based melt-back etching which destroys the crystallinity at the interface and degrades the epitaxial growth.^30−32^ The Ga-based melt-back etching process can be prevented by the introduction of an AlN layer between the GaN and the Si substrate.^32−34^ The AlN/Si heterointerfaces are characterized by abrupt and well-defined transitions.^35^

The electrically conductive Si(111) substrate is of profound interest for the ease of fabrication of a bottom contact of an NW device.^20,36^ Otherwise a complex, very high-resolution process is needed for the formation of two contacts on freestanding NWs, which is especially difficult in arrayed NW devices.^37^ In addition for III–V NWs, the lattice mismatch to Si(111) is of minor importance compared to planar layers, in which maximum performance limiting defects caused by lattice mismatch are reduced due to their outstanding property of elastic stress relaxation due to their shape.^38,39^ However, the growth of GaN NW onto a Si(111) substrate with an AlN interlayer may result in a chemically complex transition with largely unknown electrical properties, and the wide-band gap AlN interlayer may cause a high transition resistance. This results in the conflicting tendency to make the AlN layer as thick as possible to limit interdiffusion on the one hand, while at the same time making the AlN layer as thin as possible to minimize the volume of reduced conductivity. Therefore, a detailed electrical investigation with a suitable spatial resolution is necessary for the fabrication of corresponding high-performance electronic and optoelectronic devices.

In recent years, GaN NWs have already been electrically characterized using the transmission line method, which is realized with multiple nanoscale patterned electrical contacts on cleaved and then repositioned NWs.^40,41^ Accordingly, this method entails limitations for the thorough investigation of individual NWs such as the limited number of measurement points and, in particular, the determination of the NW-to-substrate-heterointerface.^42^ In contrast, the four-point measurement method using a multitip scanning tunneling microscope (MT-STM) offers the superior advantage of contacting and investigating freestanding NWs individually.^42^

We fabricated GaN NWs by polarity- and site-controlled metalorganic vapor phase epitaxy (MOVPE) growth^43^ with an AlN interlayer on n-Si(111) and characterized them individually in an upright configuration applying our MT-STM and an integrated built-in scanning electron microscope (SEM). The combination of state-of-the-art NW preparation via MOVPE and the four-tip measurement setup with completely adjustable 3D motion of the tips yields an advanced analysis for the overall electrical characterization of single NW structures and NW-to-substrate junctions. This allows us to investigate the electrical behavior of the interfaces between Si-substrates, planar AlN layers, and freestanding GaN NWs.

## Results and Discussions

We have studied the electrical behavior of freestanding n-type doped GaN NWs on n-type doped Si(111) substrates with an intermediate AlN layer. The utilization of Silane as a Si-precursor is employed to induce n-type doping in the material. The GaN NW samples are grown by a polarity- and site-controlled growth method.^43^ This method relies on prepatterning the Si(111) substrate with periodically arranged pillars of approximately 500 nm diameter,^44^ which then leads to a polarity control of the AlN layer, dependent on the growth site. The AlN interlayer is grown in pulsed mode. One cycle introduces a pulse of trimethylaluminum and a pulse of ammonia, separated by hydrogen purges. During a cycle, enough material is introduced into the reactor to grow a monolayer (ML).^44,45^ Two samples with different thicknesses of the AlN interlayer between the GaN NW and the Si substrate are characterized. First, sample A with an AlN layer consisting of 100 MLs is electrically analyzed in detail. Second, these results are compared to sample B, which includes an AlN layer consisting of 40 MLs. The samples are analyzed by utilizing the four-tip measurement setup of the MT-STM. In addition, electron-beam-induced current (EBIC) measurements are performed to reveal charge-separating contacts. Finally, the MT-STM and EBIC results are correlated with STEM images.

### Individual Characterization of GaN NW Structure by MT-STM

**Figure 1:**
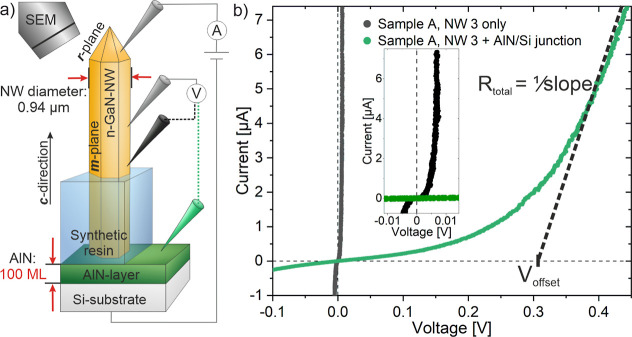
*I*–*V* characteristics of a freestanding GaN NW. (a) Schematic sketch of the measurement setup of sample A (100 MLs AlN interlayer) with two positions of the second potential measuring tip. (b) Comparison of *I*–*V* curves of sample A. The black curve depicts the electrical behavior of a NW only and the green curve shows the behavior of the NW and the NW-to-substrate junction. Inset: zoom-in of the black and green curve.

Individual upright-standing GaN NWs are characterized electrically with the help of the MT-STM. The measurement setup is depicted in Figure 1a for sample A. The NWs are partially embedded in a synthetic resin for better mechanical stability. In the first case, only the NW is analyzed by establishing the tip contacts solely on the NW sidewall, shown as the gray and black tips. In the second case, the NW-to-substrate junction is investigated. Therefore, one of the two potential measuring tips (black) is moved to the AlN layer next to the GaN NW (now shown as a green tip), where the synthetic resin coating had been removed before. The potential measuring tips have a very high input resistance of 10 TΩ to measure the potential drop between the tips current-free. Since the NWs on samples A and B are all grown with the same parameters, it can be assumed that the GaN NWs as such do not differ in their electrical properties. Therefore, such a detailed analysis is only performed on NW_A,3_.

To analyze and compare different NWs, two characteristic parameters are extracted. From the rise of the curves, outside the strongly nonlinear section, i.e. at higher currents, the reciprocal value of the slope is used to estimate the series resistance as exhibited in Figure 1b. A linear extrapolation of the high current values leads to a characteristic offset voltage (*V*_offset_). For a better comparison of the individual curves, current and voltage ranges are selected in the figures, which show only a small section of the entire curve. The procedure is shown as an example for NW 3 of sample A (NW_A,3_) in Figure 1b.

In the *I*–*V* characteristics in Figure 1b, black curve, only the GaN NW is measured. The total resistance of NW_A,3_ can be estimated as *R*_NW_ = 635 ± 50 Ω for an NW with 5 μm NW-length and a very small *V*_offset_ of around 6.5 ± 0.5 mV. The corresponding cross-sectional area *A*_total_, determined by the formula of a hexagram, is
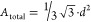
1with a measured diameter *d* from the SEM images for a total star shape.^45^ The average conductivity σ of the NW can be calculated as σ ∼ 150 (Ω cm)^−1^ for a measured cross-sectional area of 0.51 ± 0.05 μm^2^. The *I*–*V* curve shows a slight diode behavior, i.e. a charge-separating characteristic, as well as a low resistance along the NW_A,3_ of
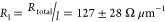
2

This is determined by the measured tip distances l of the two potential measuring tips in the recorded SEM images. This shows that the NW itself is highly conductive. The doping level can be estimated as *N*_D_ ∼ 1.6 × 10^20^ ± 3.5 × 10^19^ cm^–3^ applying the transport model^46^ as well as the Hilsum formula,^47^ and using typical values for mobility μ and impurity concentration *n*_imp_ of n-GaN,^44,45^ with μ = 820 cm^2^/(V s) and *n*_imp_ = 10^18^ cm^–3^. Due to the present high doping, the width of the depletion region of about 1 nm is negligibly small, under the assumption of ε_r_ = 10.4 and a surface potential of 0.25 eV.^48,49^ The NWs themselves are highly conductive and should not limit the efficiency of NW-based devices.

In contrast, the course of the green curve of the four-point measurement, where the second potential measuring tip is placed on the AlN layer, shows a significantly different behavior with a higher *V*_offset_ of 0.318 ± 0.001 V. Furthermore, the calculated total resistance *R*_total_ = 16.75 ± 0.14 kΩ is considerably higher than *R*_NW_ = 635 ± 50 Ω for the GaN NW only and therefore *R*_NW_ can be neglected in the measured system. Consequently, *R*_total_ determined from the green curve mainly reflects the contact behavior of the NW-to-substrate junction. Therefore, the focus of the investigation must lie on the NW-to-substrate junction. If the AlN layer acted exclusively as a region of reduced conductivity for the current transport, we would expect the AlN layer to act as a series ohmic resistor in an inversely proportional relationship to the cross-sectional area of the NW. Besides that, a thicker nonconducting layer of 100 MLs AlN compared to the thinner layer of 40 MLs should lead to an overall degradation of the conductivity of the investigated system.

**Figure 2:**
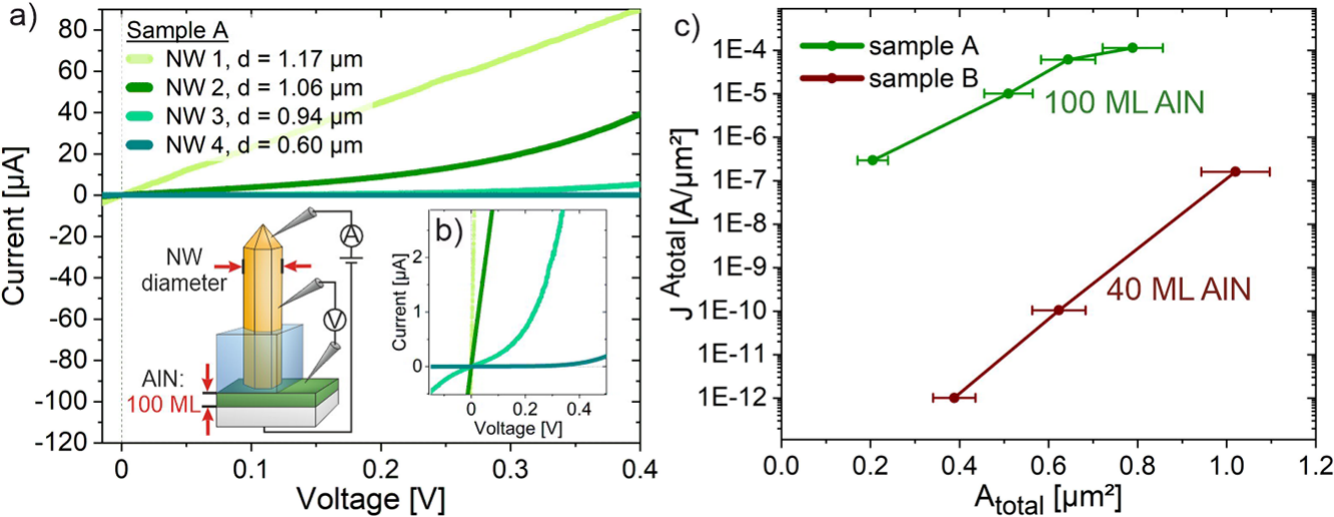
Electrical characteristics of GaN NWs. (a) *I*–*V* curves of sample A, evaluation of four NWs with different diameters and schematic sketch of measurement setup of sample A (100 MLs AlN interlayer). (b) Close up of plot (a) for better visualization of NW 3 and 4. (c) Plot of current density at *V* = 0.4 V over *A*_total_ of sample A and B with total area *A*_total_ considered for current density.

For this reason, NWs with different diameters are analyzed. The associated *I*–*V* characteristics of the GaN NWs on AlN are plotted in Figure 2a for sample A with various NW diameters (0.60 μm ≤ *d* ≤ 1.20 μm). The diameter of a NW is measured in the upper part, just below the transition of the r- and m- facets, as depicted in Figure 1a. Since the MT-STM is equipped with a built-in SEM, images of the contacted NW can be recorded to monitor its diameter *d*. It turns out that the *I*–*V* curves strongly depend on the diameter of the measured NWs. In general, we observed that an increase in the thickness of the NW was associated with a reduction in *V*_offset_ and the calculated *R*_total_, respectively, but with a stronger relation to the footprint area than expected
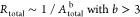
3(see Figure S1). Interestingly, NW_A,1_ shows a linear behavior with a *V*_offset_ of only 1.91 ± 0.29 mV. If NW_A,2_, which exhibits a nonlinear behavior, is compared with NW_A,1_, a very similar *R*_total_ ∼ 4.51 ± 0.01 kΩ is found, with a clear difference in *V*_offset_ ∼ 0.227 ± 0.004 V. The observation of the dependency of *V*_offset_ and *R*_total_ of NW thickness can also be recognized for sample B with 40 MLs AlN (see Figure S2), where *V*_offset_ also decreases with increasing NW diameter. *V*_offset_ is primarily related to the series resistance, which is reduced with thicker NWs and thus leads to an overall lower total resistance of the NW structure. This behavior indicates that the electrical properties of the NW-to-substrate junction change depending on the NW thickness and that the explanation of the *I*–*V* behavior cannot be attributed to a single cross-sectional area with reduced conductivity due to the variation of the cross section within the structure. Since the AlN layer thickness of sample B is significantly smaller than that of sample A, the resistance should be smaller for sample B. However, comparing NW_A,2_ from Figure 2a with NW_B,2_ from Figure S2b, which has around the same NW diameter of ∼1 μm, NW_A,2_ shows a much smaller *V*_offset_ with a difference of 3.7 V.

Calculating *R*_total_ of the measured system per cross-section area *A*_total_ of the NWs, it varies between ∼7 kΩ/μm^2^ for NW_A,2_ and ∼307 MΩ/μm^2^ for NW_B,2_ and indicates a dependency of the conductivity on the NW cross-sectional area *A*_total_. Interestingly, sample A shows better results of *R*_total_ as well as *V*_offset_ in comparison to sample B, contrary to the expectation. This becomes especially clear when the current density *J*^*A*_total_^ is plotted over the *A*_total_ of the investigated NWs, as depicted in Figure 2c. The current density is calculated from an extracted current at 0.4 V from the curves in Figures 2a and S2 (see Supporting Information). The currents flowing in sample A are several orders of magnitude higher than those flowing in sample B for the same *A*_total_. This observation can only be explained by the different thicknesses of the AlN layer between sample A and B, since the same growth conditions are applied during the MOVPE preparation for both samples. In general, the logarithmic plot shows a smaller increase with higher *A*_total_ for sample A and an approximately linear increase for sample B. Hence, the dependencies shown are due to the interplay between the lateral dimension of the NWs and the thickness of the AlN layer.

**Figure 3:**
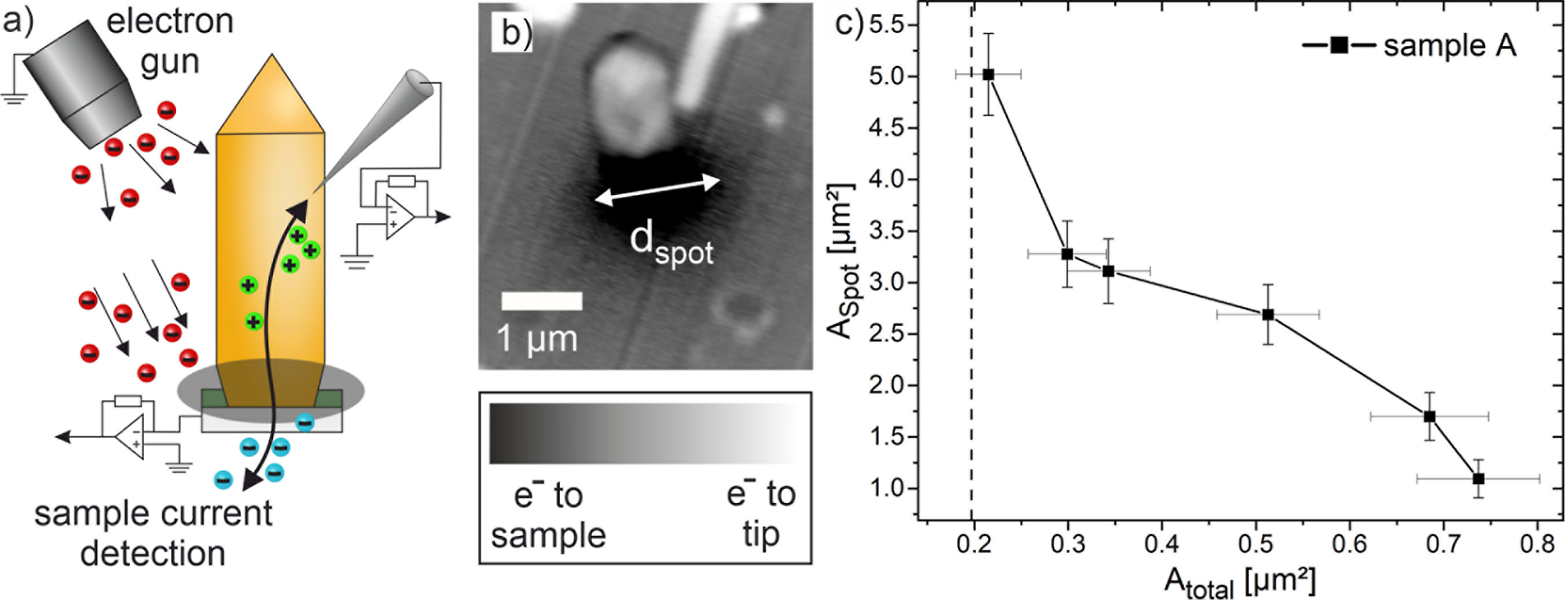
EBIC measurements at GaN NWs without embedding in synthetic resin to observe charge separating contacts of sample A. (a) Schematic illustration of EBIC measurement. (b) Tip in contact with the sidewall of the NW. Black signal indicates a high degree of charge separation at the NW base. EBIC image was taken under 22°. (c) Relationship of measured black spot-area over NW cross section area.

To investigate the dominance of the NW-to-substrate resistance in detail, sample A is reproduced without the synthetic resin to get access to the NW-to-substrate junction and it is analyzed by EBIC measurements as illustrated in Figure 3. With this method, selective charge carrier transport becomes visible when electron–hole pairs, generated locally by the incoming beam, are separated at a charge carrier selective contact.^50,51^ This results in an increased current signal measured by the transimpedance amplifier. If there is no charge carrier selective contact, electron–hole pairs can recombine and no increased current would be observed. By measuring the locally induced current in dependence on the position of the electron beam on the sample, an EBIC image can be generated. Figure 3a schematically shows the processes during the electronic injection and charge separation when a tip is brought into contact with an NW. In Figure 3b it can be seen that at the contact between the tip and the NW no additional contrast appears. This indicates, that there is no charge separation at the tip-to-semiconductor contact. Of particular interest, however, is the black spot at the location of the NW-to-substrate junction, which corresponds to a locally increased electron flow toward the substrate. This indicates that the bottom of the NW, especially the contact area between the entire NW base and the substrate, acts as a charge carrier selective contact. This becomes especially clear when the area of the black spot (*A*_spot_) is measured and plotted over *A*_total_. As the total cross-sectional area *A*_total_ increases, the spot area *A*_spot_ decreases. The EBIC signals are composed of various contributions such as the location of the electron beam, the initial background signal, and therefore the charge carrier generation rate, and their diffusion lengths.^50^ Since the same material system GaN applies to all NWs, the diffusion lengths can be assumed to be approximately constant. Accordingly, the generation of free charge carriers at the charge carrier selective contact plays a crucial role. On the one hand, in the case of thin wires in the range of 0.2 μm^2^, which corresponds to the cross-sectional area of the Si pillar, many charge carriers are separated and can contribute to the high current signal. On the other hand, charge separation with respect to *A*_spot_ is 5 times smaller for significantly thicker wires in the range of 0.75 μm^2^. This indicates that the Si pillar causes a stronger charge carrier separation than the outer areas of the wire next to the Si pillar. These EBIC measurements confirm that the NW-to-substrate junction of the freestanding vertical GaN NWs plays a decisive role in their overall electrical behavior. It also becomes clear due to the antiproportional behavior of *A*_spot_ and *A*_total_, that the AlN layer is not a simple area with reduced conductivity for current transport. To explain this behavior, we propose the following model.

### Model and Discussion

**Figure 4:**
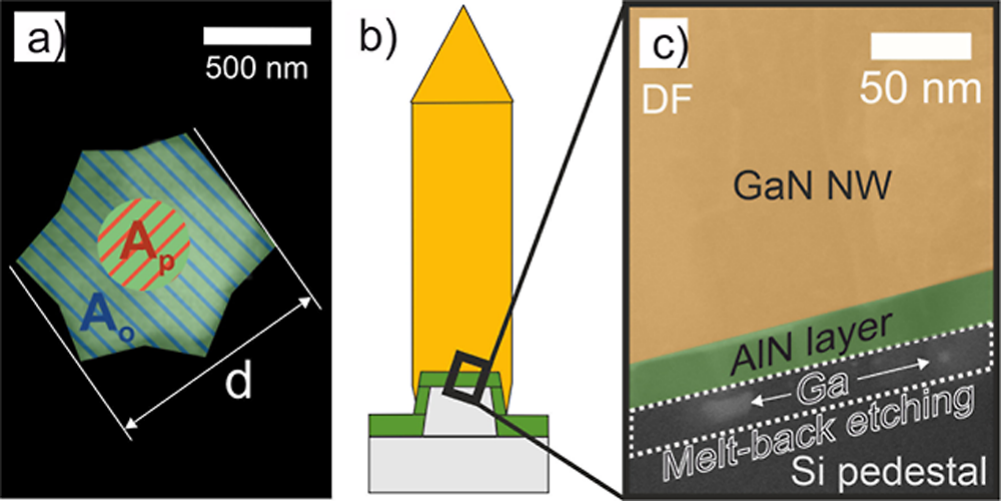
Illustration of NW configuration and dark field STEM image at the NW base. (a) Colored SEM image of one NW in top view. Contact areas between NW and substrate are marked as *A*_p_ with red stripes and *A*_o_ with blue stripes. The diameter *d* of the NW is also visualized. (b) Schematic sketch of the region taken for the STEM image. (c) Colored dark field image.

As shown by the *I*–*V* characteristics in Figure 2, the overall current behavior is essentially limited by the contact area of the GaN NW to the Si substrate. For a better understanding of the contact areas, a color-coded SEM image of an NW is shown in Figure 4a, in the top view. The contact areas between the NW and the substrate are highlighted with *A*_p_ in the red hatched area and *A*_o_ in the blue hatched area. Here and in the following, the index “p” corresponds to “Si-pillar”, and the index “o” corresponds to “overhang of the NW”.

For NWs with cross-sectional areas *A*_total_ less than or equal to the area of the Si template *A*_p_, the *I*–*V* curves show a pronounced nonlinearity. The EBIC measurements show a strong charge carrier selective transport for NWs with cross-sectional areas *A*_total_ up to area *A*_p_. By comparing samples A and B, it can be concluded that the current density is higher for a thicker AlN layer.

To better understand these characteristics, the growth process of the NW with the individual process steps, based on previous studies,^44,45^ must be examined in more detail. Since the NW growth is very complex, various processes can take place simultaneously that influence the final *I*–*V* behavior of an individual NW. Besides interdiffusion and phase segregation, dislocations, as well as diffusion of impurities can play a role. According to the literature,^33,34^ interdiffusion can be prevented by the introduction of an AlN layer between the Si-substrate and the GaN NW. For sample A with a thicker AlN layer (100 MLs), a stronger suppression of this effect is expected compared to sample B (40 MLs).

However, the melt-back etching effect can occur even when an Al interlayer is present, as shown by others.^52−54^ Therefore, we take a dark field cross-sectional STEM image of sample A (Figure 4b,c), which was previously prepared with a focused ion beam. From the STEM image, it can be observed that there are areas of increased Ga concentration in the Si pillar below the AlN layer. We conclude that interdiffusion of Ga adatoms toward the Si substrate occurs although the AlN layer is present. Since the Ga concentration in the Si pillar is so high that it is visible in the STEM image and Ga generally results in p-doping in silicon^55^ it can be assumed that local compensation of n-doping or even p-doping results. Hence, the path of the charge current through the NW-to-substrate junction is suppressed by the underlying increased Ga concentration in the Si, creating an additional unintended region of reduced electrical conductivity. The Ga adatom interdiffusion therefore creates local charge-selective junctions between the GaN-NW and Si.

Since the STEM image shows a melt-back etching effect at the NW-to-substrate interface, we assume, that the AlN layer adjacent to the NWs also allows a Ga-based melt-back etching effect. A shorter distance (40 MLs, sample B) can likely be more easily overcome by the Ga adatoms toward the Si substrate than a longer distance (100 MLs, sample A). Cross-sectional SEM images of both samples (see Figure S3) reveal a rougher surface between the NWs for sample A than B. For sample A, this implies that a fraction of the Ga adatoms remain on the AlN layer and form GaN pyramids. For sample B, however, this fraction of remaining Ga adatoms is smaller, resulting in a smoother surface. Therefore, it can be assumed that a thinner AlN layer is more susceptible to the melt-back effect for the contact area and is more likely to occur for 40 MLs of AlN interlayer than for 100 MLs.

**Figure 5:**
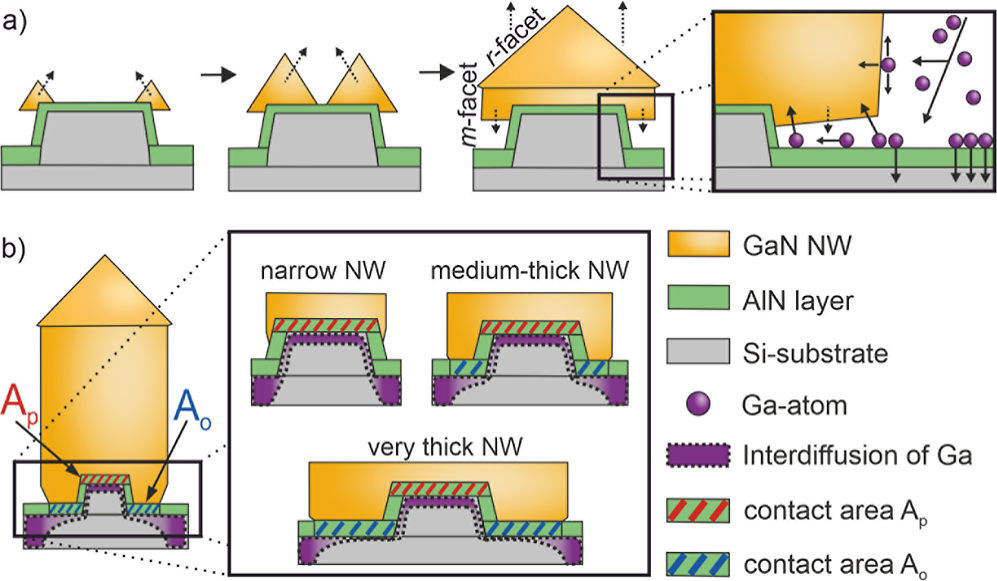
Schematic illustration of NW growth in cross section view. (a) Nucleation and formation of the growth facet applies to all NWs. (b) NW-to-substrate junction with contact area *A*_p_ (red hatched area) + *A*_o_ (blue hatched area). Inset: Size of the contact area *A*_o_ as a function of the NW cross-sectional area.

Based on these observations and assumptions a first model is proposed here: after preparation of the Si/AlN pedestals used for the polarity- and site-controlled growth,^44,45^ the next step is to deposit GaN, which initially grows as hexagonal pyramids along the top edge of sidewalls during the MOVPE process as illustrated in Figure 5a. Due to an overhang of the GaN pyramids (see Figure 5a), the AlN contact area *A*_o_ around the pedestal is partially shadowed, and fewer Ga adatoms can reach this area compared to the regions between the NWs as well as on the pedestal area. Next, these pyramids start to merge above the pedestal to form a single pyramid that defines the total NW cross-sectional area *A*_total_. Increasing the Si-to-Ga precursor ratio during the growth process leads to a silicon nitride sidewall passivation, resulting in vertical NW growth.^43^ At the same time, the NW grows downward in the −*c⃗* direction until the NW touches the AlN layer next to the pedestal region. The shadowing effect has already been described for other MOVPE processes.^56,57^ In previous growth experiments, where the growth times were varied and kept short in total, this overhang of the GaN structures is directly visible in the SEM images.^45^ After a critical lateral expansion of the GaN, Ga atoms in the gas phase can be adsorbed at the facets of the GaN NW, which contribute significantly to the growth of the GaN NW, or on the AlN layer adjacent to the pillar. During adsorption on the AlN layer, the Ga adatoms may diffuse into Si through the AlN or diffuse on the surface below the GaN overhang. The thicker the NW, the more Ga adatoms are incorporated into the growing GaN crystal. Thus, fewer Ga atoms are available for diffusion toward the Si substrate. This means that, due to the growth processes, the two contact areas differ in the time available for the interdiffusion processes. The contact area on the pedestal, labeled with *A*_p_ in Figure 5b, can be assumed to have approximately the same time for the diffusion process for different NWs. In contrast, the time available for diffusion in the region next to the pedestal below the NW, marked with *A*_o_, is longer than for area *A*_p_, but inhomogeneous in the Ga adatom distribution due to the shadowing effect of the NW itself. As a result, fewer Ga adatoms reach the region of the pedestal compared to the NW edge regions. Consequently, less interdiffusion takes place in areas close to the pedestal. Depending on the NW thickness, this shadowing effect becomes significant.

For narrow NWs, only the contact area *A*_p_ matters, because the contact area *A*_o_ does not exist. NWs with a small cross-sectional area (*A*_total_ < 0.2 μm^2^) only have the sidewalls and the terrace of the Si pillar as contact area to the Si substrate (contact area *A*_p_), as depicted in Figure 5b. For the pedestal, this means that Ga adatoms diffuse through the AlN layer into the intentionally n-doped Si, and a region with an increased Ga fraction is formed underneath the AlN layer inside the Si pedestal, compared to the rest of the substrate. Since Ga adatoms can diffuse into Si, the n-doping is (partially) compensated and results in an additional area with reduced conductivity for the charge carrier transport. For narrow NWs, this means that the Si pedestal is less n-type doped than it should be, as depicted in Figure 5b.

For medium-thickness and very thick NWs with a larger cross-sectional area *A*_total_ (>0.2 μm^2^), it can be assumed that in addition to the contact area *A*_p,_ the area *A*_o_ near the pedestal undergoes smaller changes in the doping level compared to the region toward the edge of the NW, where the Ga interdiffusion is less inhibited due to a smaller shadowing effect.

**Figure 6:**
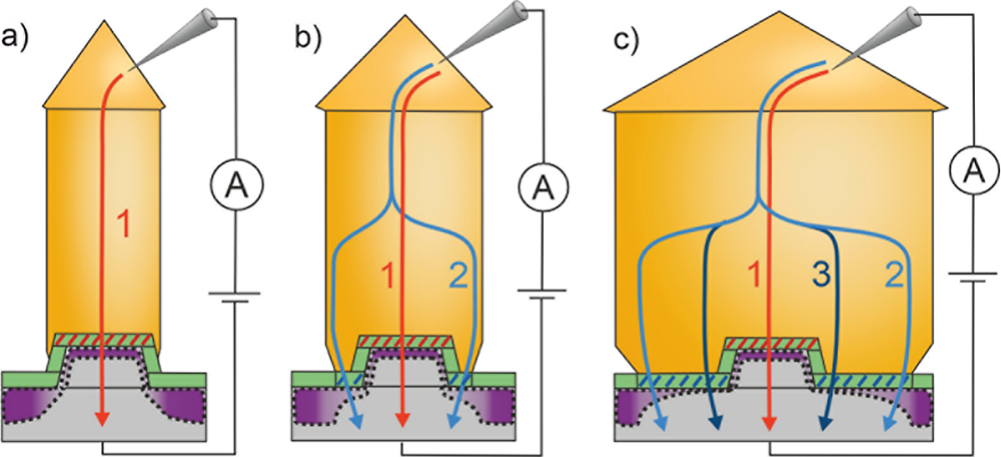
Explanation of the current paths. Schematic illustration of NW with resulting current paths in cross-sectional view. (a) Current pathways of a narrow NW. (b) Current paths of a medium-thickness NW. (c) Current paths of a very thick NW.

This assumption of the different intensities of atom-exchange processes and other defects at *A*_p_ and *A*_o_ is supported by plotting the current density over the cross-sectional area of the NW as already done in Figure 2c. The *J*^*A*_total_^ vs *A*_total_ behavior results from the fact that the partial contact area *A*_p_ has a constant cross-sectional area for all NWs, which is given by the Si pedestal preparation with *A*_p_ = 0.2 μm^2^ as the terrace cross-sectional area. Considering this, it becomes clear that the first measured value of sample A represents an NW with such a small cross-sectional area that only contact *A*_p_ is available for current transport (current path 1), as illustrated in Figure 6a. It can be assumed that the current density contribution *J*^*A*_total_^ of path 1 is determined by the size of the Si-pillar and does not change with increasing cross-sectional area *A*_total_.

An increase in current density is observed with a larger contact area *A*_total_ > 0.2 μm^2^, indicating that area *A*_o_ creates additional current paths between the NW and the substrate. As a result, the current density rises with the increasing NW cross-sectional area, as depicted in Figure 2c.

The local shadowing effect and the resulting reduced interdiffusion depend on the size of the NW, suggesting that the current pathways through *A*_o_ are also size-dependent. The shadowing effect is expected to be more pronounced near the pillar of the NW than in the outer regions. Consequently, it is anticipated that interdiffusion will be more prominent in the outer areas compared to the direct vicinity of the pillar. This implies for a medium-thickness NW, path 2 is present in addition to path 1. However, due to the medium-thickness of the NW, path 2 has a generally low shadowing effect and still clear interdiffusion. Consequently, path 2 is preferred, but only marginally better than path 1 in terms of the total current density. The potential current paths for a medium-thickness NW are illustrated in Figure 6b.

Due to the inhomogeneous influence of the shadowing effect, the reduced interdiffusion at the vicinity of the pillar is noticeable for very thick NWs with *A*_total_ > 0.51 μm^2^, which is expressed by the drastic change of the slope of the current density in Figure 2c. This small area of *A*_o_ exhibits a better conductivity, represented by path 3 in Figure 6c. Paths 1 and 2 become less significant compared to path 3 with increasing NW diameter, especially for *d* > 1 μm, where a less pronounced increase in the current density is observed in Figure 2c.

Comparing samples A and B, it is obvious that atomic exchange processes occur in both samples, although in sample B only NWs with larger cross-sectional areas *A*_total_ ≥ 0.2 μm^2^ are measured. Thus, no data with smaller cross-sectional areas are available. In general, the measured current densities of sample B differ strongly from those of sample A by several orders of magnitude. The different current densities between samples A and B can be explained by the assumption that a thinner AlN layer suppresses the interdiffusion processes less than a thicker AlN layer. Since these interdiffusion processes are more strongly suppressed by the thicker AlN layers of sample A, the current density of sample A is significantly higher for the measured cross-sectional NW areas. Thus, current path 1 exhibits a reduced current density in sample B compared to sample A even at small NW cross sections.

However, with increasing NW diameter, the current density increases for both samples, but the increase is stronger for sample B. The reason for this is the shadowing effect in combination with the different thicknesses of the AlN layer. Current path 3 becomes more important, especially for very thick NWs. Due to the shadowing, almost no melt-back etching occurs near the Si pillar, creating a current path in this area (path 3) that undergoes less or no atom-exchange processes for both samples. In this particular region, the AlN layer would act as a potential barrier, and a thinner AlN layer would be more beneficial for the overall electrical behavior. This would explain a stronger increase in the current density with increasing cross-sectional area *A*_total_ for sample B compared to A. Therefore, there could also be a flattening of the current density increase for such thick NWs outside of the measured range of *A*_total_ for sample B.

## Conclusion

We have investigated the electronic properties of patterned, freestanding n-GaN NWs on Si(111) substrates with an AlN interlayer grown by a polarity- and site-controlled growth method applying an MT-STM. The introduction of the AlN layer between the GaN and Si-substrate results in a high interface resistance. While the GaN NWs themselves exhibit a very low resistance of 127 ± 28 Ω/μm along the NW, however, the overall performance of the grown structure is strongly influenced by the NW-to-substrate contact. It is found that the effects on the *I*–*V* curves cannot be explained by a simple area with reduced conductivity. Rather, a complex system exists at the NW-to-substrate junction. Several aspects such as atom exchange processes, dislocations, phase segregation, and impurities strongly reduce the current in the investigated bias range. The effects of Ga adatom shadowing below the GaN NW, next to the Si pedestal, and their interdiffusion are crucial in this context. A model for the current paths and their dependence on growth mechanisms is suggested and supported by STEM images and EBIC measurements. In addition, samples with different AlN interlayer thicknesses are electrically characterized. It is found that a thicker AlN layer better suppresses the interdiffusion processes and provides a better overall electrical behavior up to a certain NW cross-sectional area of about 1.4 μm^2^. Therefore, the NW base is critical for the overall electrical performance of the bottom-up grown GaN NWs on Si. With this knowledge of utilizing the shadowing effects and optimizing the AlN thickness, conductive backside contacts can be fabricated for GaN nanowires on Si. A fully linear *I*–*V* characteristic is demonstrated up to a preliminary current density of *J* ≥ 30 A/cm^2^, which is well suited for medium power density devices such as LEDs or solar cells.

## Experimental Section/Methods

### Sample Preparation

The NWs are grown by polarity- and site-controlled growth.^43^ Nanoimprint technology is used to p the aforementioned pillars on the whole 2″ Si(111) substrate. The substrates are oxidized in a plasma step before loading into the reactor. In a hydrogen desorption bake step, site-controlled removal of oxygen is achieved leading to a site-dependent growth of the AlN layer. The different properties of the AlN layer lead to an enhanced nucleation of GaN at these Si-pillars. N-doped NWs with a height of approximately 5 μm were fabricated. Details about the growth process are reported elsewhere.^43^ After growth, the NWs are completely covered in a photoactive resin. Using UV light exposure and developer liquid, the resin is removed from the top until a height of approximately 3 to 4 μm. This leaves the top 1 to 2 μm of the NWs accessible, while enhancing their mechanical stability for the measurements.

### Electrical Characterization by MT-STM

By the nondestructive measurement principle of the MT-STM, single, freestanding NWs can be precisely electrically characterized utilizing four individually movable tungsten tips.^58−60^ Through the control of the additionally installed built-in SEM, each tip can be positioned by piezo elements at a desired contact point at the NW or planar layers.^61^ For a four-point measurement, an electric current is induced by applying a voltage between the measuring tip at the top of the NW and the back contact. Two additional tips are spatially positioned, one at the sidewall of the NW underneath the current measuring tip and the other at the AlN layer as potential measuring tips. This unique setup allows the exclusion of contact and series resistors from the measurement, which is crucial for accessing charge separation junctions. For each measurement tip, a transimpedance amplifier and specially designed electronics can be used to select between high-impedance voltage and low-impedance current measurements.^62^ Therefore, due to the high input resistance of 10 TΩ at the potential measuring tips, it can be assumed that the potential is measured current-free and that there is no lateral current flow within the AlN layer as a result of the low conductivity of the AlN layer,^36,63^ which might affect the measured *I*–*V* curve.

As suggested by Koch et al.,^64^ the measurement results comply with all the necessary steps for a reliable tip-based *I*–*V* measurement. Only measurements without hysteresis are considered. The SEM is blanked during the measurement to prevent a contribution from the SEM-induced current.

## Supporting Information Available

Supporting Information_MT-STM on GaN NW (PDF)

## Abbreviations

MT-STM: multitip scanning tunnelling microscope
SEM: scanning electron microscope
EBIC: electron-beam induced current
MOVPE: metalorganic vapor phase epitaxy
NW: nanowire
ML: monolayer
