# Supplementary material for: Tracking Charge Carrier Paths in Freestanding GaN/AlN Nanowires on Si(111)
Source: ACS Appl Mater Interfaces. 2024 Sep 19;16(39):52780–8. doi: 10.1021/acsami.4c10179 (PMC11450776; doi:10.1021/acsami.4c10179)
Supplement: Supplementary file 1 — am4c10179_si_001.pdf [file am4c10179_si_001.pdf]

Supporting Information

Tracking charge carrier paths in freestanding GaN/AlN nanowires on Si(111)

Juliane Koch[1]*, Patrick Häuser*[2]*, Peter Kleinschmidt*[1]*, Werner Prost*[2]*, Nils Weimann*[2] *and Thomas Hannappel*[1*]

[1] Department of Mathematics and Natural Science, Institute for Physics, Fundamentals of Energy Materials, Ilmenau University of Technology, Ilmenau 98693, Germany

[2] Components for High Frequency Electronics (BHE), University of Duisburg-Essen, Duisburg 47057, Germany

[*] corresponding author's email address: thomas.hannappel@tu-ilmenau.de

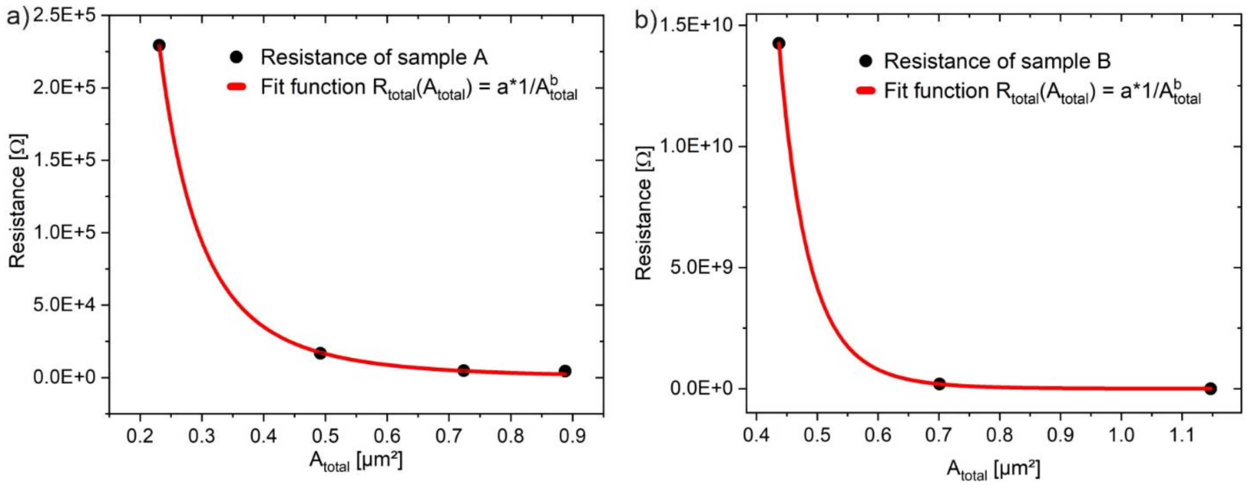

Figure S1. $R_{total} – A_{total}$ characteristics of a) sample A with fitting parameters a = 1,529.00 Ωm and b= 3.42; and b) of sample B with fitting parameters a = 7,490,566.00 Ωm and b = 9.12.

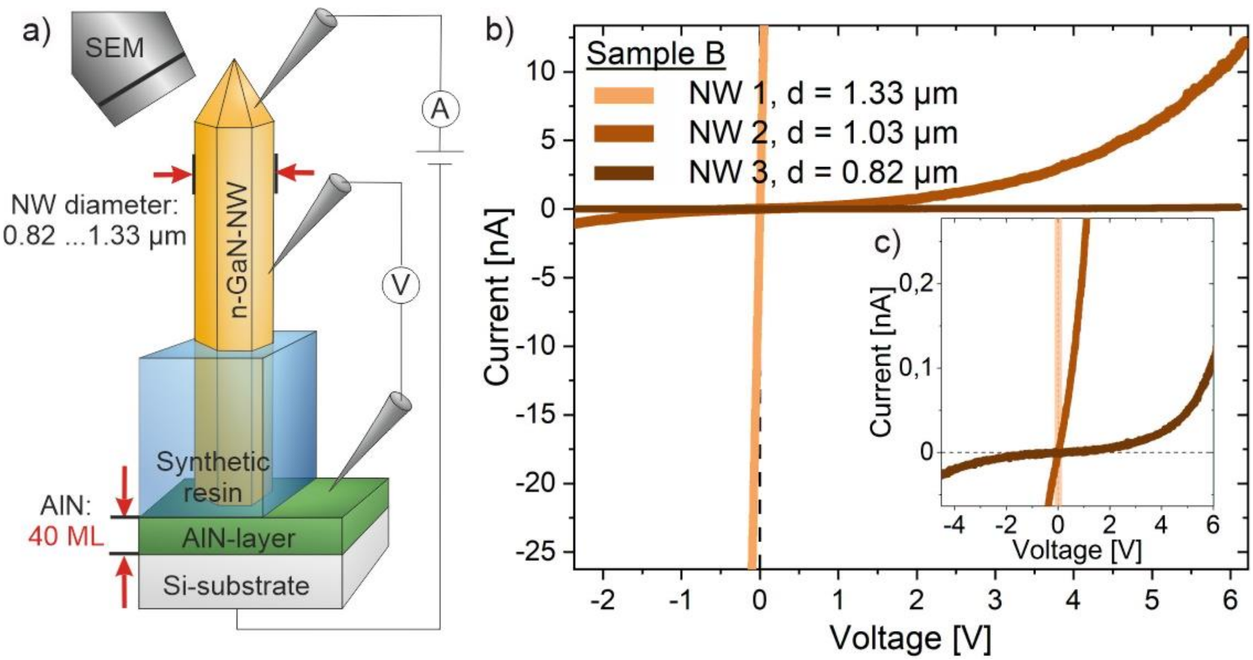

Figure S2. *I-V* characteristics of GaN NWs. a) Schematic sketch of measurement setup of sample B (40 ML AlN planar layer). b) *I-V* curves of sample B, analyzation of three NWs with different diameters. c) Close-up of plot b) for better visualization of NW 3.

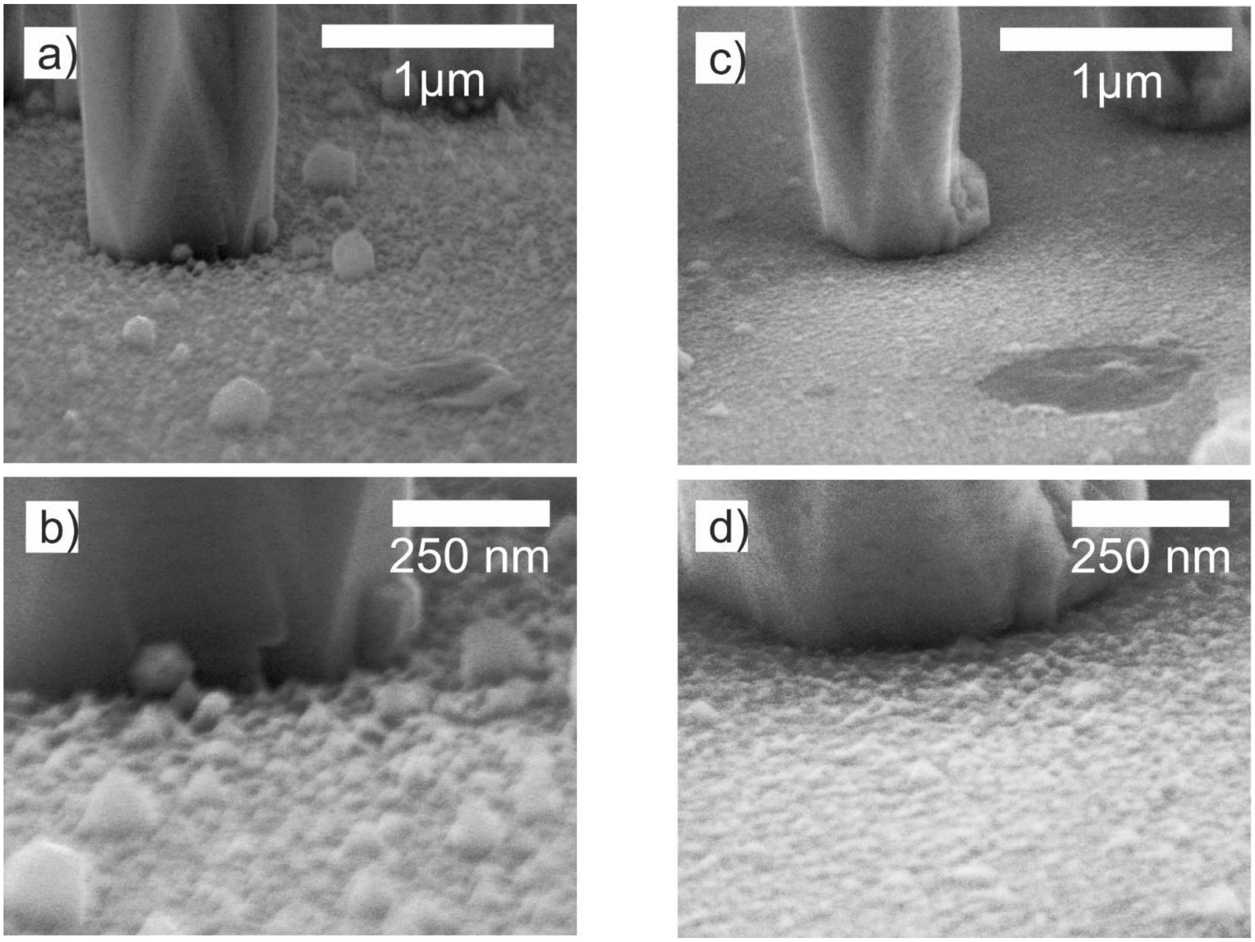

Figure S3. Cross-sectional SEM images of region between GaN NWs. a) Sample A (100 ML AlN planar layer). b) Close-up image of sample A. c) Sample B (40 ML AlN planar layer). d) Close-up image of sample B.
